# Factors Influencing Health Care Technology Acceptance in Older Adults Based on the Technology Acceptance Model and the Unified Theory of Acceptance and Use of Technology: Meta-Analysis

**DOI:** 10.2196/65269

**Published:** 2025-03-28

**Authors:** Hyo Jun Yang, Ji-Hyun Lee, Wonjae Lee

**Affiliations:** 1 Graduate School of Culture Technology Korea Advanced Institute of Science and Technology Daejeon Republic of Korea

**Keywords:** technology adoption, older adults, health care technology, technology acceptance model, unified theory of acceptance and use of technology, meta-analysis

## Abstract

**Background:**

The technology acceptance model (TAM) and the unified theory of acceptance and use of technology (UTAUT) are widely used to examine health care technology acceptance among older adults. However, existing literature exhibits considerable heterogeneity, making it difficult to determine consistent predictors of acceptance and behavior.

**Objective:**

We aimed to (1) determine the influence of perceived usefulness (PU), perceived ease of use (PEOU), and social influence (SI) on the behavioral intention (BI) to use health care technology among older adults and (2) assess the moderating effects of age, gender, geographic region, type of health care technology, and presence of visual demonstrations.

**Methods:**

A systematic search was conducted across Google Scholar, Web of Science, Scopus, IEEE Xplore, and ProQuest databases on March 15, 2024, following PRISMA (Preferred Reporting Items for Systematic Reviews and Meta-Analyses) guidelines. Of the 1167 initially identified studies, 41 studies (11,574 participants; mean age 67.58, SD 4.76 years; and female:male ratio=2.00) met the inclusion criteria. The studies comprised 12 mobile health, 12 online or telemedicine, 9 wearable, and 8 home or institution hardware investigations, with 23 studies from Asia, 7 from Europe, 7 from African-Islamic regions, and 4 from the United States. Studies were eligible if they used the TAM or UTAUT, examined health care technology adoption among older adults, and reported zero-order correlations. Two independent reviewers screened studies, extracted data, and assessed methodological quality using the Newcastle-Ottawa Scale, evaluating selection, comparability, and outcome assessment with 34% (14/41) of studies rated as *good* quality and 66% (27/41) as *satisfactory*.

**Results:**

Random-effects meta-analysis revealed significant positive correlations for PU-BI (*r*=0.607, 95% CI 0.543-0.665; *P*<.001), PEOU-BI (*r*=0.525, 95% CI 0.462-0.583; *P*<.001), and SI-BI (*r*=0.551, 95% CI 0.468-0.624; *P*<.001). High heterogeneity was observed across studies (*I*²=95.9%, 93.6%, and 95.3% for PU-BI, PEOU-BI, and SI-BI, respectively). Moderator analyses revealed significant differences based on geographic region for PEOU-BI (*Q*=8.27; *P*=.04), with strongest effects in Europe (*r*=0.628) and weakest in African-Islamic regions (*r*=0.480). Technology type significantly moderated PU-BI (*Q*=8.08; *P*=.04) and SI-BI (*Q*=14.75; *P*=.002), with home or institutional hardware showing the strongest effects (PU-BI: *r*=0.736; SI-BI: *r*=0.690). Visual demonstrations significantly enhanced PU-BI (*r*=0.706 vs *r*=0.554; *Q*=4.24; *P*=.04) and SI-BI relationships (*r*=0.670 vs *r*=0.492; *Q*=4.38; *P*=.04). Age and gender showed no significant moderating effects.

**Conclusions:**

The findings indicate that PU, PEOU, and SI significantly impact the acceptance of health care technology among older adults, with heterogeneity influenced by geographic region, type of technology, and presence of visual demonstrations. This suggests that tailored strategies for different types of technology and the use of visual demonstrations are important for enhancing adoption rates. Limitations include varying definitions of *older adults* across studies and the use of correlation coefficients rather than controlled effect sizes. Results should therefore be interpreted within specific contexts and populations.

## Introduction

### Background

According to the United Nations, the number of people aged ≥65 years is projected to be 1.6 billion in 2050, which is double of what it was in 2021 [1], and the number of persons aged ≥80 years is projected to be 143 million in 2050, triple of what it was in 2019 [2]. This demographic shift raises significant concerns for the escalating burden on health care systems and associated financial implications. Advanced age is often associated with a higher prevalence of chronic illnesses and age-related conditions, necessitating increased medical attention and resources [3,4].

Technology can play a role in supporting older adults in health care by allowing quicker information and communication, preventing the development of chronic conditions, and monitoring health conditions [5]. At the same time, they can also reduce caregiver burden, leading to cheaper and better-quality care [6]. However, despite the potential benefits offered by these advancements, their widespread adoption remains limited, primarily due to ambivalence among older adults toward technology acceptance [7]. Therefore, identifying the factors that affect the acceptance of technology for older adults is one of the most important research needs to support older adults’ use of technology [8].

However, a key issue is that the current literature that aims to understand the acceptance of health care technology for older adults exhibits significant heterogeneity, with diverse studies yielding varying effects and strengths of predictors [9]. To explain, the technology acceptance model (TAM) [10] and the unified theory of acceptance and use of technology (UTAUT) [11] are commonly used by scholars to understand technology in the context of health [12] and were applied in research of different types of health care technology and their acceptance by older adults [13-15]. TAM uses perceived usefulness (PU) and perceived ease of use (PEOU), with the subsequent TAM2 adding subjective norm, while UTAUT identifies performance expectancy (PE), effort expectancy (EE), social influence (SI), and facilitating conditions as core determinants to explain behavioral intention (BI). The constructs of TAM and UTAUT can be combined due to their conceptual similarities and complementary nature and previous studies have used the findings of both models in meta-analysis [16]. PU and PE both reflect the belief that using technology will enhance performance, while PEOU and EE indicate the perceived effort required to use the technology. SI, akin to subjective norm in TAM2, addresses the influence of other important factors on technology use. Both models use the constructs to explain BI, the intention to adopt technology.

To elaborate the heterogeneity of the TAM and UTAUT literature, studies have shown that older adults are more likely to accept technology that meets their needs and expectations [17,18]. However, the impact of PU on technology acceptance varies for health care technology. Li et al [19] found that PU had very little impact on the BI to adopt a remote health management service for older adults while Mahmood and Lee [20] reported a high influence of PU for health monitoring wearable technology. For older adults, ease of use is crucial because physical and cognitive abilities affect the acceptance and use of technology [21,22]. However, studies are conflicting. Wu et al [23] identified a high effect for the acceptance of medical self-service terminals, but Khan et al [24] observed a low effect for mobile health (mHealth) services. Similarly, for SI, while it is understood SI can significantly affect older adults’ technology adoption, particularly those from their children, friends, and professional caregivers [25], heterogeneity exists in the current literature. Koo et al [26] detected a high effect of SI for the acceptance of a personalized health care service app while Wong et al [27] determined that SI had no effect on the use of the internet for health information in one of the 2 models used in the study.

Such heterogeneity makes it difficult to interpret results because the inconsistent findings across studies prevent a clear, unified understanding of the effects of PU, PEOU, and SI on BI for health care technology acceptance among older adults. This study aimed to perform a meta-analysis to systematically aggregate and analyze these diverse results, providing a more robust and comprehensive assessment of the factors influencing health care technology adoption in this population and the characteristics of the primary studies that have moderating effects. Similar research, such as the quantitative meta-analysis by Chong et al [16], provided an expansive study into the TAM and UTAUT literature on health care information technologies but did not focus on older adults and was limited to a specific type of health care technology, while Ma et al [28] focused on literature that applied the 2 models for older adults without specifying the type of technology. Therefore, a meta-analysis that specifically targets older adults and their acceptance of different types of health care technology is needed to provide a more defined analysis of the current literature.

### Objectives

To ensure a clearer interpretation of the current literature regarding the acceptance of health care technology for older adults, this study aimed to (1) synthesize the sample size–weighted average of the PU-BI, PEOU-BI, and SI-BI relationships for the current literature that used TAM or UTAUT to examine the acceptance of health care technology for older adults and (2) identify sources of systematic heterogeneity by analyzing the sample and methodology characteristics of the primary studies that moderate the PU-BI, PEOU-BI, and SI-BI relationships.

## Methods

### Literature Search

While traditional systematic review frameworks such as patient, intervention, comparison, and outcome or sample, phenomenon of interest, design, evaluation, research type were considered, these structures did not optimally align with our research focus on technology acceptance behavior, particularly as elements such as interventions or comparisons were not directly applicable to our context. Instead, we developed a focused search strategy specifically designed to capture studies examining TAM, UTAUT, and their associated constructs in the context of older adults’ health care technology acceptance.

The systematic search was conducted in 5 databases (Google Scholar, Web of Science, Scopus, IEEE Xplore, and ProQuest) on March 15, 2024. Google Scholar was included for its ability to capture a broad range of interdisciplinary studies beyond purely clinical research, aligning with the scope of this study. While we initially searched PubMed as it is a prioritized database for clinical studies, it yielded fewer relevant results, leading us to expand our search to additional databases. Testing confirmed that the PubMed results were already captured by the selected databases, affirming the adequacy of our chosen sources.

A combination of 3 groups of word strings was developed using logical operators (AND/OR) as follows: (1) age-related keywords: *older adult* OR *elderly* OR *ageing* OR *aging*, (2) theoretical framework keywords: *unified theory of acceptance and use of technology* OR *UTAUT* OR *technology acceptance model* OR *TAM* OR *acceptance* OR *adoption* OR *intention*, and (3) context of use keywords: *health* OR *healthcare* OR *well-being* OR *gerontechnology.*

The search terms were collaboratively developed by 3 reviewers (HJY, JHL, and WJL) and refined through iterative rapid searches across the selected databases. These rapid searches were conducted to evaluate the effectiveness of the search terms, ensuring they comprehensively captured relevant studies while minimizing irrelevant results. The final search terms were chosen to align with the study’s focus on TAM and UTAUT frameworks for health care technology acceptance among older adults. The full search terms and detailed search strategy are provided in Multimedia Appendices 1 and 2. The number of articles identified, screened, eligible, and included were recorded according to the PRISMA (Preferred Reporting Items for Systematic Reviews and Meta-Analyses) statement (Multimedia Appendix 2).

### Eligibility Criteria

In total, 2 reviewers independently screened the titles and abstracts to identify studies eligible for full-text screening and subsequently conducted the full-text screening of the selected studies. Any disagreements during this process were resolved through a majority vote, with input from a third researcher to ensure consensus. To ensure that the selected studies were directly relevant to the research question, a rigorous inclusion criteria were applied during the full-text screening as described in Textbox 1.

**Inclusion and exclusion criteria for meta-analysis of health care technology acceptance for older adults.**

**Inclusion criteria**

- Article type: peer-reviewed journal articles, conference papers, and dissertations
- Language: English-language publications
- Theoretical framework: studies using technology acceptance model (including perceived usefulness [PU] and perceived ease of use [PEOU]) or the unified theory of acceptance and use of technology (including performance expectancy and effort expectancy)
- Technology context: studies focusing on health care technology, including mobile health, wearable devices, telemedicine, and home-based health care systems
- Study population: older adults (generally aged ≥50 years) or studies explicitly targeting older populations
- Outcome measures: studies reporting zero-order correlations between PU-behavioral intention (BI), PEOU-BI, and social influence-BI, or similar constructs
- Study characteristics: studies with a single, clearly defined characteristic, such as one geographic region or one technology type

**Exclusion criteria**

- Article type: editorials, opinion pieces, book chapters, and nonpeer-reviewed sources
- Language: non-English publications
- Theoretical framework: studies that do not use technology acceptance model or the unified theory of acceptance and use of technology as the primary theoretical framework
- Technology context: studies examining non–health care–related technologies or general technology acceptance without health care relevance
- Study population: studies that do not specify an older adult population or focus on general populations
- Outcome measures: studies that do not provide statistical correlation data relevant to the analysis
- Study characteristics: studies that mix multiple regions or technology types without providing separate analyses

If a study’s sample included 2 or more of the same characteristics (eg, participants from both Asia and Europe and survey regarding multiple types of technology), it was not included. Theses and dissertations were involved as well to reduce the chance of publication bias. If a study met the criteria but did not report the zero-order correlations, an email was sent to the corresponding author to request the information. The inclusion and exclusion criteria are shown in Textbox 1.

### Data Extraction

The coding procedure for the primary studies’ characteristics was designed to ensure the extraction of the required data. The 2 researchers separately extracted the information of the primary studies into a Microsoft Excel spreadsheet. If any disagreements arose, the third researcher, who is an experienced statistician, was involved for a resolution. The following information was coded:

1. Required data, including the author’s name, publication year, sample size, and correlation coefficient of PU-BI, PEOU-BI, and SI-BI
2. Continuous variables for moderator analysis, including mean age of the sample and gender ratio
3. Categorical variables for moderator analysis, including region the study where it was conducted, type of health care technology, and presence of technology demonstration

The mean age was calculated using frequency counts in age-stratified data or directly extracted from the studies if provided. Geographic region was coded based on the location of the sample collection. The gender ratio was determined by dividing the number of male participants by the number of female participants. Technology domains were categorized into 4 distinct groups: mHealth (eg, mHealth apps and mHealth services), wearable (eg, smart clothing and smart watches), online and telemedicine (eg, online health platforms and remote consultations), and home and institutional health hardware (eg, fall monitoring systems and self-service health kiosks). The presence of technology demonstrations in the studies, whether through a video or live demonstration just before the survey or experiment, was recorded as a binary variable.

### Quality Assessment

In total, 2 reviewers independently assessed the methodological quality of studies using the Newcastle-Ottawa Scale. The Newcastle-Ottawa Scale uses a star system to evaluate the methodological quality of studies. The adapted cross-sectional tool assigns up to 8 points across 3 domains: (1) selection of study groups (up to 4 points), (2) comparability of the groups based on age and sex (up to 2 points), and (3) assessment of outcomes (up to 2 points). For this study, it was adapted to properly evaluate TAM- and UTAUT-related studies. The ascertainment of exposure was evaluated based on the use of surveys, with a score of 1 if surveys were used and 0 if no information was given. The comparability domain assessed control for age and sex, with 2 points if both were controlled, 1 point if one was controlled, and 0 points if neither were controlled. The assessment of outcomes was based on the use of validated surveys and the reporting of Cronbach α, with 2 points if both were provided, 1 point if only the validated survey was used, and 0 points if neither were described.

### Data Synthesis and Analysis

Data were synthesized according to the review objectives.

#### Objective 1: Synthesize the Sample Size–Weighted Average of the PU-BI, PEOU-BI, and SI-BI Relationships for the Current Literature

To pool the effect sizes, random-effects analysis was used to calculate the sample size–weighted correlation of the PU-BI, PEOU-BI, and SI-BI relationships. To pool the effect sizes, random-effects analysis was used instead of a fixed effects analysis because it accounts for variance that is not just from sampling error but differences in population, methodology, and setting [29]. While the fixed effects model offers a more intuitive method of assigning weights that is solely based on sample size of the study [30], the variations of TAM and UTAUT studies make the random-effects model more suitable. It was conducted using R (R Foundation for Statistical Computing) with the *meta* package which contains functions that make it easy to run different types of meta-analyses [31]. To address potential publication bias, funnel plots were generated through the *meta* package, providing a visual means to detect systematic bias in the meta-analysis. The trim-and-fill method was then applied to adjust for any detected bias, ensuring the pooled effect size was representative and robust. This method is able to account for missing effects that may arise from publication bias to correct for small-study effects for the pooled effect sizes [32]. In summary, the study reported the sample size–weighted average of the 3 pairwise relationships, their 95% CIs, the adjusted weighted averages, and the adjusted 95% CIs.

#### Objective 2: Identify Sources of Systematic Heterogeneity by Analyzing the Characteristics of the Primary Studies That Moderate the PU-BI, PEOU-BI, and SI-BI Relationships

Meta-regression was used to explore the impact of the continuous moderators, such as age and gender, on the 3 pairwise relationships. The significance level for the beta coefficients was considered at *P*<.05. Subgroup analysis was used for the categorical moderators region, technology domain, and presence of visual demonstration on the 3 pairwise relationships. A *Q* test was conducted to measure the heterogeneity between the subgroups and the difference was considered significant at *P*<.05.

## Results

### Study Selection

**Figure 1:**
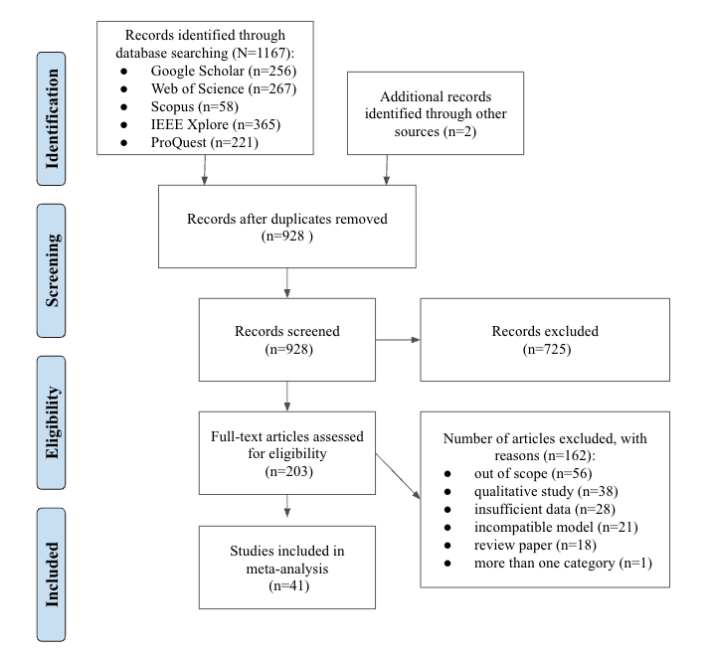
PRISMA flow diagram of evidence search and selection.

The literature search process yielded 1167 studies (Figure 1). After the removal of duplicates, 928 studies remained for the full-text screening. After assessing the full-text articles for eligibility, the resulting number of studies included in the meta-analysis was 41. There were 2 studies that met the eligibility criteria after additional information was provided by the corresponding author.

### Study Characteristics

The summary of the characteristics of the studies is included in Table 1. The total sample size of the primary studies was 11,574. Of the 41 studies, 33 (80%) explicitly included the mean age of participants, with an overall mean of 67.58 (SD 4.76) years and a range of 57.86 to 82.1 years. In total, 5 (12%) studies provided an exact age range: ≥60 years [19,24,33], 50 to 65 years [34], and ≥50 years [35]. The remaining 3 (7%) studies [36-38], while not specifying a mean age, used consistent but indefinite terminology, such as *elderly* or *older adults* in their title, abstract, introduction, or methodology. Therefore, it was assumed that the sample of the 3 (7%) studies was consistent with the focus of this research and fell within the broader age range represented by the 33 (80%) studies that reported mean ages. In total, 36 (88%) studies reported the gender distribution of their samples, the total ratio being 2.00, with more female participants than male. For each technology type, there were 9 (22%) studies for wearable, 12 (29%) for mHealth, 12 (29%) for online or telemedicine, and 8 (19%) for home or institution hardware. In total, 23 (56%) studies were conducted in Asia (China, Taiwan, and Korea), 7 (17%) in Europe, 4 (10%) in the United States, and 7 (17%) in African-Islamic countries (Bangladesh, Saudi Arabia, and Pakistan). The description of the studies is included in Table 2.

**Table 1:** Characteristics of 41 primary studies included in meta-analysis.

Author and year	Sample size, n	Age (y), mean	Gender ratio	Technology type	Region	Visual demonstration
Akhter and Hossain [39], 2022	112	57.86	0.87	mHealth^a^	African-Islamic	No
Alsswey and Al-Samarraie [40], 2019	81	63.48	0.35	mHealth	African-Islamic	No
Boontarig et al [36], 2012	31	—^b^	—	Online or telemedicine	Asia	No
Cimperman et al [41], 2016	400	61.13	1.03	Online or telemedicine	Europe	No
Cristescu et al [37], 2022	750	—	—	Wearable	Europe	No
Diño and de Guzman [38], 2014	82	—	2.57	Online or telemedicine	Asia	No
Etemad-Sajadi and Gomes Dos Santos [42], 2019	213	82.1	2.60	Home or institution hardware	Europe	No
Greer and Abel [43], 2022	30	66.3	—	mHealth	United States	No
Hoque and Sorwar [44], 2017	274	68.06	0.51	mHealth	African-Islamic	No
Hsiao and Tang [45], 2015	338	67.12	1.08	Wearable	Asia	Yes
Jeng et al [46], 2022	166	67.81	1.24	Wearable	Asia	Yes
Khan et al [24], 2022	286	—	0.74	mHealth	African-Islamic	Yes
Kim et al [47], 2022	269	76.1	1.10	Home or institution hardware	Asia	No
Koo et al [26], 2023	477	64.31	0.73	mHealth	Asia	Yes
Li et al [48], 2018	146	67.41	0.78	Wearable	Asia	Yes
Li et al [49], 2021	353	70.38	1.37	Online or telemedicine	Asia	No
Li et al [19], 2023	402	—	0.61	Online or telemedicine	Asia	No
Lu and Tsai-Lin [13], 2024	510	75.13	4.15	Online or telemedicine	Asia	No
Ma and Luo [50], 2023	1318	63.98	0.89	mHealth	Asia	No
Mahmood and Lee [20], 2021	376	70	1.56	Wearable	United States	Yes
Mascret and Temprado [51], 2023	230	66.61	2.03	Home or institution hardware	Europe	Yes
Mascret et al [52], 2020	271	73.69	1.71	Home or institution hardware	Europe	Yes
Maswadi et al [15], 2022	486	70.80	1.59	Home or institution hardware	African-Islamic	No
Mukherjee [53], 2021	200	70.75	1.53	Online or telemedicine	United States	No
Pal et al [54], 2018	239	67.14	0.52	Home or institution hardware	Asia	No
Palas et al [14], 2022	493	66.33	0.18	mHealth	African-Islamic	No
Pate [34], 2022	128	—	—	Wearable	United States	No
Pywell [55], 2021	313	63.89	1.52	mHealth	Europe	No
Quaosar et al [33], 2018	245	—	—	mHealth	African-Islamic	No
Ren and Zhou [56], 2023	200	68.83	1.33	Online or telemedicine	Asia	No
Rój [57], 2022	400	64.5	1.17	Online or telemedicine	Europe	Yes
Talukder et al [58], 2020	325	67.89	0.56	Wearable	Asia	No
Techatraiphum et al [35], 2016	45	—	—	Online or telemedicine	Asia	No
Tsai et al [59], 2020	81	69.7	0.89	Wearable	Asia	Yes
Tu and Liu [60], 2021	487	67.11	1.99	Online or telemedicine	Asia	No
Wang et al [61], 2023	365	67.31	1.28	mHealth	Asia	Yes
Wong et al [27], 2014	98	64.93	1	Online or telemedicine	Asia	No
Wu et al [23], 2023	78	61.78	1.69	Home or institution hardware	Asia	Yes
Xu et al [62], 2022	51	68.96	1.32	Home or institution hardware	Asia	Yes
Zhang [63], 2023	55	59.9	0.67	mHealth	Asia	No
Zin et al [64], 2023	170	68.85	1.27	Wearable	Asia	No

^a^mHealth: mobile health.

^b^Not applicable.

**Table 2:** Description of the characteristics of the included primary studies.

Characteristics			Statistical results
Studies, n			41
Total sample size			11,574
Age (y), mean (SD)			67.58 (4.76)
**Gender ratio (female:male)**			
	Mean (SD)	1.26 (4.52)	
	>1, n (%)	22 (61)	
	≤1, n (%)	14 (39)	
**Technology type, n (%)**			
	Wearable	9 (22)	
	Mobile health	12 (29)	
	Online or telemedicine	12 (29)	
	Home or institution hardware	8 (19)	
**Region, n (%)**			
	Asia	23 (56)	
	Europe	7 (17)	
	United States	4 (10)	
	African-Islamic	7 (17)	

### Analysis Characteristics

Of the 41 studies, most did not end with just a correlation analysis but conducted more in-depth statistical methods, such as structural equation modeling and multivariate regression. Although all the correlation coefficients were positive, certain studies reported path coefficients that were not statistically significant or did not have the exact pathway in their final model. The rate at which the correlation coefficient represents the final reporting of the studies is given in Table 3. For the PU-BI relationship, 41 correlation coefficients were extracted from a correlation matrix given in the study. Of the 41 correlation coefficients, 31 (76%) had PU-BI path analysis, 27 (66%) of which were significant and positive with a rate of 87% (27/31). For the PEOU-BI relationship, 41 correlation coefficients were extracted. Of the 41 studies, 28 (68%) included the PEOU-BI path analysis, 21 (51%) of which were positive and significant with a rate of 75% (21/28). For the SI-BI relationship, 28 correlation coefficients were extracted. Of the 28 studies, 21 (75%) included the SI-BI path analysis, 16 (57%) of which were positive and significant with a rate of 76% (16/21).

**Table 3:** Representativeness of final path analysis by correlation coefficient of pairwise relationships.

Pairwise relationship	Frequency of correlation analysis, n	Frequency of path analysis, n (%)	Frequency of significant path analysis, n (%)	Rate of significant path analysis (%)
Perceived usefulness–BI^a^	41	31 (76)	27 (66)	87
Perceived ease of use–BI	41	28 (68)	21 (51)	75
Social influence–BI	28	21 (75)	16 (57)	76

^a^BI: behavioral intention.

### Quality Assessment

#### Overview

Of the 41 studies, the majority (n=27, 66%) were assessed to be of *satisfactory* quality. A few studies (14/41, 34%) were considered of *good* quality. No studies were assessed as *very good* quality or *unsatisfactory* quality. Quality assessment results (Table 4) for these studies are summarized in the following sections.

**Table 4:** Newcastle-Ottawa Scale for quality assessment of the included studies.

Study	Selection					Comparability		Outcome			Quality score^a^
	Representativeness	Sample size	Nonrespondents	Ascertainment of the exposure	Confounding factors controlled		Assessment of outcome		Statistical test		
Akhter and Hossain [39], 2022	0	1	Unsure	1	0		1		1	Satisfactory	
Alsswey and Al-Samarraie [40], 2020	0	Unsure	1	1	0		1		1	Satisfactory	
Boontarig et al [36], 2012	Unsure	0	Unsure	1	0		1		1	Satisfactory	
Cimperman et al [41], 2016	1	1	Unsure	1	0		1		1	Good	
Cristescu et al [37], 2022	Unsure	1	Unsure	1	0		1		1	Satisfactory	
Diño and de Guzman [38], 2014	0	0	Unsure	1	0		1		1	Satisfactory	
Etemad-Sajadi and Gomes Dos Santos [42], 2019	0	0	0	1	0		1		1	Satisfactory	
Greer and Abel [43], 2022	0	0	Unsure	1	0		1		1	Satisfactory	
Hoque and Sorwar [44], 2017	1	1	1	1	0		1		1	Good	
Hsiao and Tang [45], 2015	1	0	1	1	0		1		1	Good	
Jeng et al [46], 2022	0	0	1	1	0		1		1	Satisfactory	
Khan et al [24], 2022	0	0	1	1	0		1		1	Satisfactory	
Kim et al [47], 2022	0	0	1	1	0		1		1	Satisfactory	
Koo et al [26], 2023	0	1	1	1	0		1		1	Good	
Li et al [48], 2019	0	0	Unsure	1	0		1		1	Satisfactory	
Li et al [49], 2021	0	1	1	1	0		1		1	Good	
Li et al [19], 2023	0	1	Unsure	1	0		1		1	Satisfactory	
Lu and Tsai-Lin [13]	1	1	1	1	0		1		1	Good	
Ma and Luo [50], 2023	0	1	0	1	0		1		1	Satisfactory	
Mahmood & Lee [20], 2021	1	0	Unsure	1	0		1		1	Satisfactory	
Mascret and Temprado [51], 2023	0	0	Unsure	1	0		1		1	Satisfactory	
Mascret et al [52], 2020	1	1	0	1	0		1		1	Good	
Maswadi et al [15], 2022	1	1	Unsure	1	0		1		1	Good	
Mukherjee [53], 2021	0	1	Unsure	1	0		1		1	Satisfactory	
Pal et al [54], 2018	0	0	1	1	0		1		1	Satisfactory	
Palas et al [14], 2022	1	1	1	1	0		1		1	Good	
Pate [34], 2022	0	1	1	1	0		1		1	Good	
Pywell [55], 2021	0	1	1	1	0		1		1	Good	
Quaosar et al [33], 2018	0	0	1	1	0		1		1	Satisfactory	
Ren and Zhou [56], 2023	1	0	1	1	0		1		1	Good	
Rój [57], 2022	1	1	Unsure	1	0		1		1	Good	
Talukder et al [58], 2020	0	0	1	1	0		1		1	Satisfactory	
Techatraiphum et al [35], 2016	0	0	1	1	0		1		1	Satisfactory	
Tsai et al [59], 2020	0	0	Unsure	1	0		1		1	Satisfactory	
Tu and Liu [60], 2021	0	1	1	1	0		1		1	Good	
Wang et al [61], 2023	Unsure	1	1	1	0		1		1	Good	
Wong et al [27], 2014	Unsure	Unsure	Unsure	1	0		1		1	Satisfactory	
Wu et al [23], 2023	0	Unsure	Unsure	1	0		1		1	Satisfactory	
Xu et al [62], 2022	0	Unsure	Unsure	1	0		1		1	Satisfactory	
Zhang [63], 2023	0	1	0	1	0		1		1	Satisfactory	
Zin et al [64], 2023	0	1	Unsure	1	0		1		1	Satisfactory	

^a^Very good studies: 7 to 8 points, good studies: 5 to 6 points, satisfactory studies: 3 to 4 points, and unsatisfactory studies: 0 to 2 points.

#### Selection

In the selection domain, the studies demonstrated varied results. A small number of studies (10/41, 24%) had representative samples, with most studies (37/41, 90%) applying convenience or purposive sampling to gain an unrepresentative sample. Around half of the studies (20/41, 49%) provided adequate sample size, either with a sample size over 400 or by providing justification for their size. Less than half (19/41, 46%) studies had a response rate >80%, while 18 (44%) studies did not provide sufficient details, and 4 (10%) studies had a response rate <80%. All studies were given 1 star for the ascertainment of exposure as the information was obtained through surveys.

#### Comparability

Regarding comparability, all studies received 0 stars in this domain. This is because, while some studies did control for age or gender through structural equation modeling or multivariate regression, the information used in this analysis was based on correlation coefficients before these controls were applied.

#### Outcome

In the outcome domain, all studies were given the maximum score of 2 stars for the assessment of the outcome. Specifically, all 41 studies used validated questionnaires from previous TAM and UTAUT studies and reported Cronbach α values, which demonstrated the reliability of the measures. This consistent use of validated measures and reliability reporting supports the overall credibility of the outcome data.

### Effect Sizes and Publication Bias Adjustment

**Figure 2:**
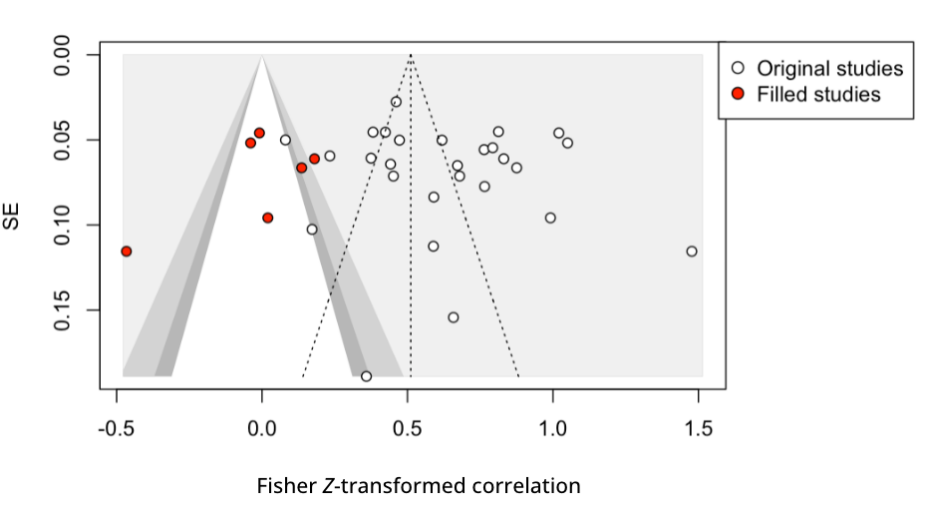
Funnel plot of social influence–behavioral intention relationship after trim and fill.

The weighted average of the correlations of the 3 pairwise relationships, PU-BI (*r*=0.607, 95% CI 0.543-0.665; *P*<.001), PEOU-BI (*r*=0.525, 95% CI 0.462-0.583; *P*<.001), and SI-BI (*r*=0.551, 95% CI 0.468-0.624; *P*<.001), was calculated using the random-effects model. The CI of each effect indicated a positive relationship for the intention to accept technology. The heterogeneity was calculated through a *Q* test and an *I*^2^ test for PU-BI (*Q*_40_=973.77; *P*<.001; I^2^=95.9%), PEOU-BI (*Q*_40_=626.95; *P*<.001; I^2^=93.6%), and SI-BI (*Q*_27_=580.59; *P*<.001; I^2^=95.3%), all of which showed a high degree of heterogeneity. To test the possibility of publication bias, the funnel plot method was used to detect any asymmetry. If detected, the trim-and-fill method was used to adjust the weighted average of the effect by filling in additional points to maintain symmetry. Among the 3 relationships, SI-BI required 6 additional points as shown in Figure 2, while the PU-BI and SI-BI required none. The weighted average of the effect sizes and the adjustments after the trim-and-fill are reported in Table 5. The table summarizes the results of the 3 pairwise relationships through quantitative synthesis by confirming that each pair has a positive association, indicating that the intention to accept health care technology is dependent on the usefulness, ease of use, and SI.

**Table 5:** Weighted correlation of pairwise relationships of studies included.

Pairwise relationship	Total sample size, n	Weighted correlation, *r* (95% CI)	Adjusted weighted correlation, *r* (adjusted 95% CI)
Perceived usefulness–BI^a^	11,574	0.607 (0.542-0.665)	0.607 (0.542-0.665)
Perceived ease of use–BI	11,574	0.525 (0.462-0.583)	0.525 (0.462-0.583)
Social influence–BI	8264	0.559 (0.476-0.632)	0.471 (0.363-0.566)

^a^BI: behavioral intention.

### Moderator Analysis

#### Age

Of the 41 studies, 33 (80%) were included in the meta-regression with the sample age as its coefficient. One relationship, PU-BI (β=0.000; *P*=.99), was positively associated with the mean age of the sample while PEOU-BI (β=−0.126; *P*=.19) and SI-BI (β=−0.017; *P*=.35) were negatively associated with age. However, the significance test showed that the results for the 3 relationships were not significant.

#### Gender

While 35 primary studies reported the number of female and male participants, 1 study [43] that involved 29 females and 1 male was removed as it was deemed an outlier. Therefore, the remaining 34 studies were included in the meta-regression with the female-to-male ratio as its coefficient. PU-BI (β=0.126, *P*=.07), PEOU-BI (β=0.037, *P*=.56), and SI-BI (β=0.105, *P*=.34), were positively associated with the proportions of female participants over the male participants. However, the significance test proved none of the relationships to be significant.

#### Geographic Region

**Figure 3:**
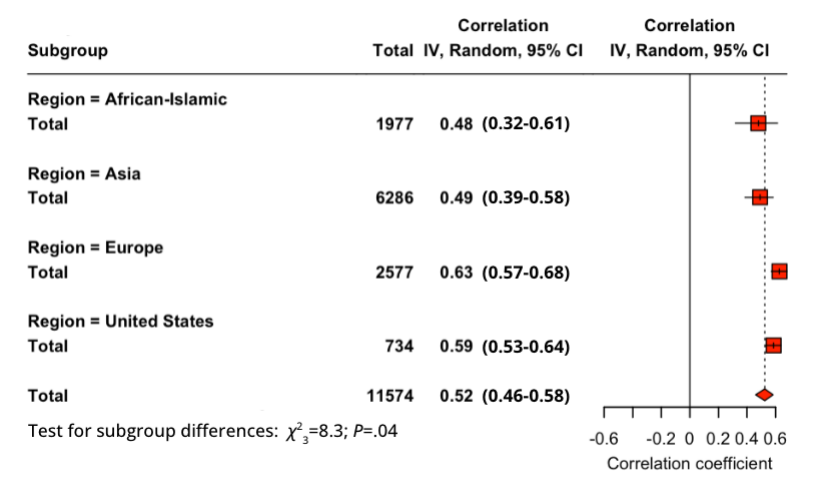
Subgroup analysis of region for perceived ease of use–behavioral intention relationship.

A subgroup analysis of the geographic regions was conducted to report the effect size of each relationship for each region, the United States, Europe, Asia, and African- Islamic. From highest to lowest in correlation for PU-BI (*Q*_3_=6.3; *P*=.10), the order was the United States (*r*=0.713), Europe (*r*=0.712), Asia (*r*=0.572), and African-Islamic (*r*=0.529). For PEOU-BI (*Q*_3_=8.27; *P*=.04), the order was Europe (*r*=0.628), the United States (*r*=0.587), Asia (*r*=0.492), and African-Islamic (*r*=0.480). For SI-BI (*Q*_3_=2.63; *P*=.45), the order was the United States (*r*=0.700), Europe (*r*=0.602), Asia (*r*=0.548), and African-Islamic (*r*=0.495). After conducting a *Q* test, it was observed that only the PEOU-BI relationship (Figure 3) had a difference in the subgroups large enough to be considered significant.

#### Technology Type

**Figure 4:**
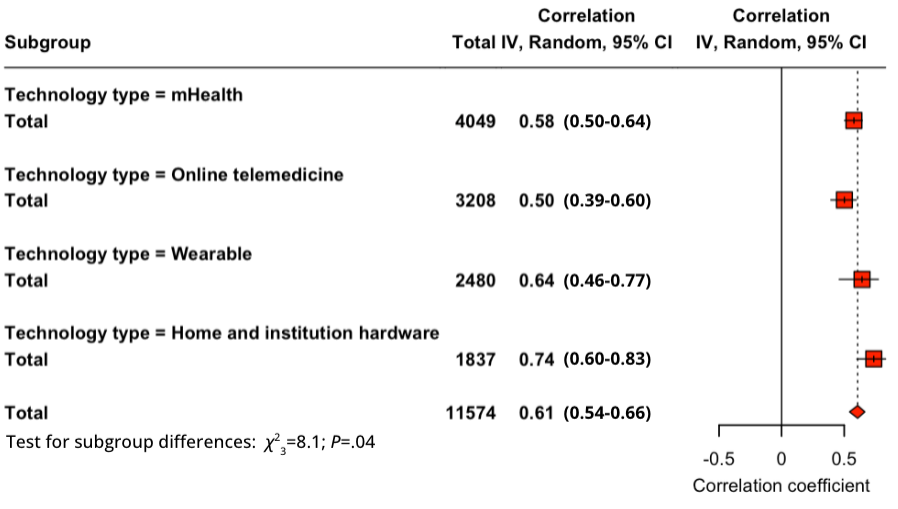
Subgroup analysis of health care technology type for perceived usefulness–behavioral intention relationship. mHealth: mobile health.

**Figure 5:**
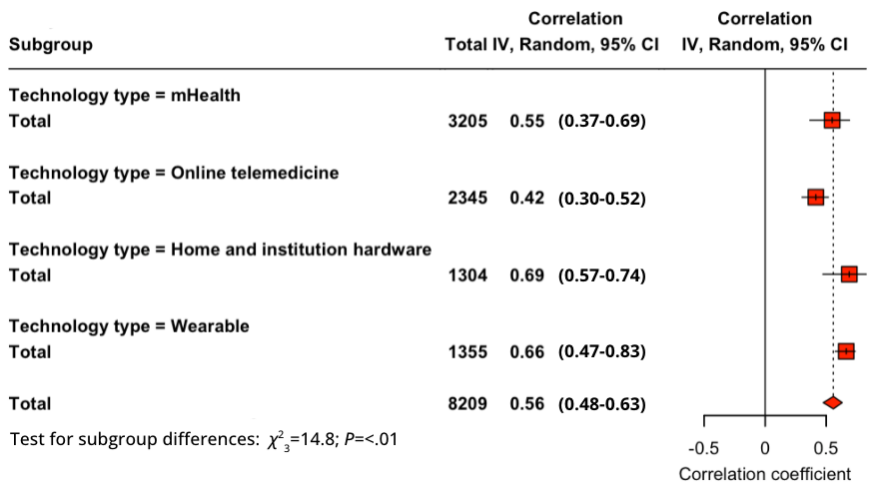
Subgroup analysis of health care technology type for social influence–behavioral intention relationship. mHealth: mobile health.

All studies were involved in a subgroup analysis of the technology type, which were divided into wearable, mHealth, online or telehealth, and home or institutional. For PU-BI (*Q*_3_=8.08; *P*=.04), the order from highest to lowest in correlation was home or institutional (*r*=0.736), wearable (*r*=0.642), mHealth (*r*=0.578), and online or telehealth (*r*=0.501). For PEOU-BI (*Q*_3_=4.15; *P*=.25), it was home or institutional (*r*=0.641), mHealth (*r*=0.510), online or telehealth (*r*=0.489), and wearable (*r*=0.467). For SI-BI (*Q*_3_=14.75; *P*=.002), the order was home or institutional (*r*=0.690), wearable (*r*=0.664), mHealth (*r*=0.550), and online or telehealth (*r*=0.415). The *Q* test for the PU-BI (Figure 4) and SI-BI (Figure 5) proved the differences in the subgroups to be significant but not for PEOU-BI.

#### Visual Demonstration

**Figure 6:**
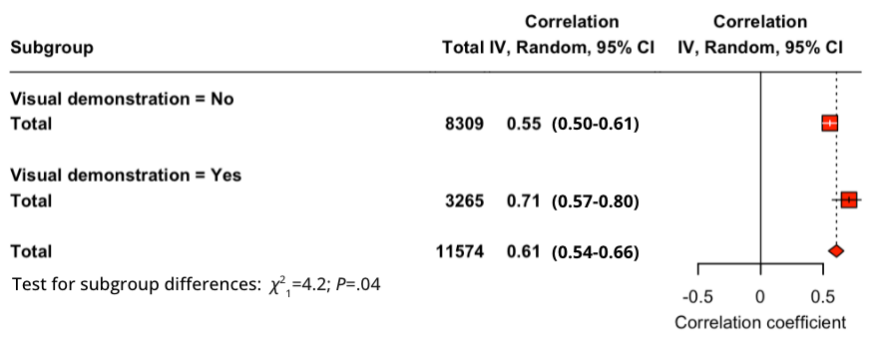
Subgroup analysis of presence of visual demonstration for perceived usefulness–behavioral intention relationship.

**Figure 7:**
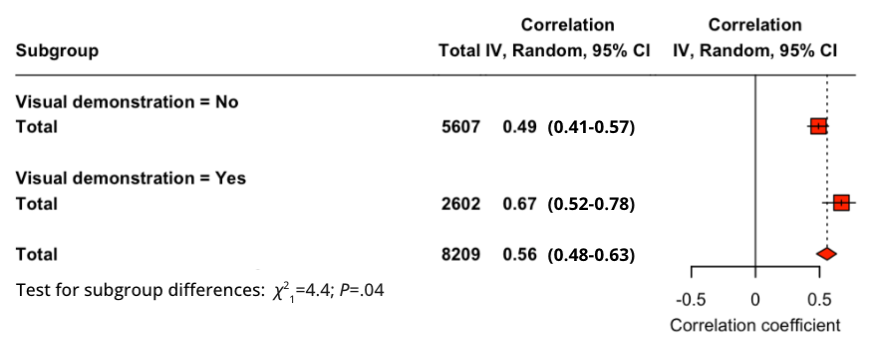
Subgroup analysis of presence of visual demonstration for social influence–behavioral intention relationship.

A subgroup analysis was conducted by dividing the studies into 2 groups, one that involved a visual demonstration of the technology before the survey, and one without. For the relationship PU-BI (*Q*_1_=4.24; *P*=.04), studies that provided a visual demonstration (*r*=0.706) had a higher effect size compared to studies that did not (*r*=0.554). For PEOU-BI (*Q*_1_=0.16; *P*=.69), studies that provided a visual demonstration (*r*=0.535) displayed a lower effect compared to studies that did not (*r*=0.501). Finally, for SI-BI (*Q*_1_=4.38; *P*=.04), studies that provided a visual demonstration (*r*=0.670) displayed a higher effect than studies without (*r*=0.492). For PU-BI (Figure 6) and SI-BI (Figure 7), the *Q* test proved the significance of their difference but not for PEOU-BI.

## Discussion

### Principal Findings

This meta-analysis aimed to address 2 primary objectives: synthesizing the current evidence on health care technology acceptance among older adults and identifying potential sources of heterogeneity. The findings revealed significant positive correlations for all 3 key relationships: PU-BI (*r*=0.607), PEOU-BI (*r*=0.525), and SI-BI (*r*=0.551). Further analysis identified significant moderating effects of geographic region on PEOU-BI, technology type on both PU-BI and SI-BI, and visual demonstrations on PU-BI and SI-BI relationships, while age and gender showed no significant moderating effects.

### Overall Effect Sizes

The pooled results extend our understanding of how older adults accept health care technology. While a separate study noted a lower correlation between PU and BI for health care technology compared to other types of technology [65], our meta-analysis revealed that PU maintains a strong influence on older adults’ intention to use health care technology. The significant PEOU-BI relationship aligns with the understanding that older adults may face cognitive and physical challenges [66,67], making ease of use particularly crucial for technology adoption and accessibility [68]. The strong SI-BI relationship confirms the substantial influence of family members, friends, and caregivers on health care technology adoption decisions [65], providing empirical support for the importance of social networks in technology acceptance among older adults.

### Moderator Analysis

#### Age

The analysis revealed that the relationships between the constructs and BI did not vary with age. This is in line with the results from the study by Ma et al [28] that found a lack of moderating effect of age for acceptance of various types of technology, suggesting that a deeper look into health care technology reports the same results [28]. In addition, meta-analysis by Hauk et al [69] reported that age has a negative effect on TAM constructs unless the technology in question addresses the needs of older individuals, explaining the absence of a moderating effect on health care technology. Such results provide a new way of understanding the effect of age for technology acceptance that differs from the age stereotypes that assume a negative effect of age.

#### Gender

The gender analysis did not show any significant effect on the relationships studied, suggesting that gender does not significantly impact the acceptance decisions related to health care technology. A review paper [70] concluded that the influence of gender on technology adoption depends on the context and type of technology, implying that gender effects may not be relevant in the context of health care technology. It is important to note that the primary studies included in this meta-analysis had more female participants than male, which could introduce potential bias. Consequently, the results related to gender should be interpreted with caution, and future research should aim for a more balanced sample to ensure comprehensive understanding of health care technology acceptance among older adults.

#### Geographic Region

Subgroup analysis by geographic region revealed that for PU-BI, the United States and Europe displayed the highest correlations, followed by Asia and African-Islamic regions, indicating that PU is more strongly related to BI in Western cultures. For PEOU-BI, Europe showed the highest correlation, followed by the United States, Asia, and African-Islamic regions. This is in line with the findings of McCoy et al [71] that reported that the PU-BI and PEOU-BI relationship tends to be weaker for countries that have a higher power distance. The countries in the Asia category (China, Taiwan, and Korea) and African-Islamic category (Bangladesh, Saudi Arabia, and Pakistan) report much higher power distance.

#### Technology Type

Home or institutional hardware exhibited the highest correlations for both PU and PEOU. These technologies, which include devices such as smart home systems and health kiosks, are inherently designed to improve quality of life, making their usefulness and user-friendliness paramount to their adoption [47,72]. In contrast, online or telehealth technologies showed the lowest correlations across PU and SI. This suggests factors such as privacy or trust as identified in other studies [73,74] may be more critical determinants of acceptance. Another important finding is the high correlation of SI-BI and low correlation of PEOU-BI for wearable technology. This suggests that social proof and endorsements could be powerful tools in promoting wearable technology within this demographic. A possible reason for this is because wearable technology is not perceived as just a health care technology but as a fashion accessory [75], which is why production quality and social value are important factors [58].

#### Visual Demonstration

The significant differences observed in the studies that included visual demonstrations emphasize the crucial role of reducing perceived risk in consumer theory for the acceptance of health care technology by older adults. Perceived social risk and physical risk play an important role when acquiring information about new technology [76] but visual demonstrations can reduce the abstractness and uncertainty surrounding new health care technology by providing clear, tangible evidence of its functionality. Specifically for older adults, perception of automated vehicles, such as its PU, for older adults improved after exposure to a simulator and a demonstration in an automated shuttle [77]. Similarly, a practical engagement may solidify the constructs of PU, PEOU, and SI by directly showcasing exactly how the technology works and its practical benefits, thus making other unidentified variables influencing BI less significant.

### Limitations

This meta-analysis has several important limitations that should be considered when interpreting the findings. First, the main source of analysis, which is the correlation coefficient of the TAM constructs, were used instead of the finalized path coefficient of the primary studies. While this ensures the comparability and synthesis of the weighted averages of the effect sizes, it may not fully represent the results of the primary studies as the control variables are removed in the correlation analysis. Second, the substantial heterogeneity observed across the included studies, as reflected in the high *I*² values (PU-BI: 95.9%, PEOU-BI: 93.6%, and SI-BI: 95.3%), represents a significant limitation. This heterogeneity likely stems from differences in study populations, geographic regions, types of health care technology, and methodologies. Although the use of a random-effects model mitigated the impact of this variability by accounting for between-study differences, it remains challenging to generalize the findings to all older adult populations or health care technologies. Researchers should interpret the pooled effect sizes cautiously, as they represent averages across diverse study contexts rather than universally consistent effects. Third, the definition of *older adults* varied across the included studies, with most defining participants as >60 years of age [14,15,26,40,44-50,56-58,60,61], while others set the range at 55 years [13,27,54,63,64], 50 years [23,39,41,43,55,59], or ≥65 years [20,52,53,62]. Some studies did not clearly specify participant ages, relying instead on general descriptors such as *older adults* or *elderly*. Although the meta-regression suggested that mean age did not significantly influence the results, this variation reflects a potential limitation in how age was operationalized. This lack of consistency should be considered when interpreting the findings, and future research should aim for more standardized age definitions. Finally, the use of different methods across studies to collect information has noteworthy limitations. Although most studies obtained their data through web-based surveys, other methods such as face-to-face distribution, telephone, and mobile surveys were used, creating the possibility of the mode effect.

### Conclusions

This meta-analysis provides a framework to understand and interpret the heterogeneous nature of health care technology acceptance among older adults. Rather than viewing inconsistent findings in previous literature as methodological weaknesses, our results suggest they may reflect genuine variations in acceptance patterns across different contexts and technologies. These insights have important implications for both research and practice. Future studies should carefully consider and report contextual factors that our analysis has identified as significant moderators, while also addressing current methodological limitations through standardized age definitions and balanced sampling approaches. This could lead to a more comprehensive understanding of technology acceptance among older adults and support the development of more effective implementation strategies for health care technology—an increasingly important consideration as health care systems worldwide adapt to serve older adult populations.

## Appendix

Multimedia Appendix 1 Complete search term and additional figures.

Multimedia Appendix 2 PRISMA checklist.

## Abbreviations

BI: behavioral intention
mHealth: mobile health
PEOU: perceived ease of use
PRISMA: Preferred Reporting Items for Systematic Reviews and Meta-Analyses
PU: perceived usefulness
SI: social influence
TAM: technology acceptance model
UTAUT: unified theory of acceptance and use of technology

## Data Availability

The datasets generated or analyzed during this study are available in the Figshare repository [78].

## References

[1] World social report 2023: leaving no one behind in an ageing world. United Nations (UN).

[2] World population ageing 2019. United Nations (UN).

[3] Czaja S, Beach S, Charness N, Schulz R, Sixsmith A, Gutman G (2013). Older adults and the adoption of healthcare technology: opportunities and challenges. Technologies for Active Aging.

[4] van den Akker M, Buntinx F, Metsemakers JF, Roos S, Knottnerus J (1998). Multimorbidity in general practice: prevalence, incidence, and determinants of co-occurring chronic and recurrent diseases. J Clin Epidemiol.

[5] Czaja SJ (2017). The potential role of technology in supporting older adults. Public Policy Aging Rep.

[6] Wang J, Carroll D, Peck M, Myneni S, Gong Y (2016). Mobile and wearable technology needs for aging in place: perspectives from older adults and their caregivers and providers. Stud Health Technol Inform.

[7] Wu YH, Damnée S, Kerhervé H, Ware C, Rigaud AS (2015). Bridging the digital divide in older adults: a study from an initiative to inform older adults about new technologies. Clin Interv Aging.

[8] Rogers WA, Mitzner TL, Sanford JA (2014). A research framework to guide design of technologies for successful aging with disabilities. Gerontechnology.

[9] Legris P, Ingham J, Collerette P (2003). Why do people use information technology? A critical review of the technology acceptance model. Inf Manag.

[10] Davis FD (1989). Perceived usefulness, perceived ease of use, and user acceptance of information technology. MIS Q.

[11] Venkatesh V, Morris MG, Davis GB, Davis FD (2003). User acceptance of information technology: toward a unified view. MIS Q.

[12] Rouidi M, Elouadi AE, Hamdoune A, Choujtani K, Chati A (2022). TAM-UTAUT and the acceptance of remote healthcare technologies by healthcare professionals: a systematic review. Inform Med Unlocked.

[13] Lu CC, Tsai-Lin TF (2024). Are older adults special in adopting public eHealth service initiatives? The modified model of UTAUT. Sage Open.

[14] Palas JU, Sorwar G, Hoque MR, Sivabalan A (2022). Factors influencing the elderly's adoption of mHealth: an empirical study using extended UTAUT2 model. BMC Med Inform Decis Mak.

[15] Maswadi K, Ghani NA, Hamid S (2022). Factors influencing the elderly's behavioural intention to use smart home technologies in Saudi Arabia. PLoS One.

[16] Chong AY, Blut M, Zheng S (2022). Factors influencing the acceptance of healthcare information technologies: a meta-analysis. Inf Manag.

[17] Kim S, Gajos KZ, Muller M, Grosz BJ (2016). Acceptance of mobile technology by older adults: a preliminary study. Proceedings of the 18th International Conference on Human-Computer Interaction with Mobile Devices and Services.

[18] Nägle S, Schmidt L (2012). Computer acceptance of older adults. Work.

[19] Li W, Gui J, Luo X, Yang J, Zhang T, Tang Q (2023). Determinants of intention with remote health management service among urban older adults: a unified theory of acceptance and use of technology perspectivegy perspective. Front Public Health.

[20] Mahmood N, Lee Y (2021). Factors influencing older adults’ acceptance of health monitoring smart clothing. Fam Consum Sci Res J.

[21] Czaja SJ, Charness N, Fisk AD, Hertzog C, Nair SN, Rogers WA, Sharit J (2006). Factors predicting the use of technology: findings from the Center for Research and Education on Aging and Technology Enhancement (CREATE). Psychol Aging.

[22] Farshchian BA, Dahl Y (2015). The role of ICT in addressing the challenges of age-related falls: a research agenda based on a systematic mapping of the literature. Pers Ubiquit Comput.

[23] Wu Q, Huang L, Zong J (2023). User interface characteristics influencing medical self-service terminals behavioral intention and acceptance by Chinese elderly: an empirical examination based on an extended UTAUT model. Sustainability.

[24] Khan T, Khan KD, Azhar MS, Shah SN, Uddin MM, Khan TH (2021). Mobile health services and the elderly: assessing the determinants of technology adoption readiness in Pakistan. J Public Aff.

[25] Peek ST, Wouters EJ, van Hoof J, Luijkx KG, Boeije HR, Vrijhoef HJ (2014). Factors influencing acceptance of technology for aging in place: a systematic review. Int J Med Inform.

[26] Koo JH, Park YH, Kang DR (2023). Factors predicting older people's acceptance of a personalized health care service app and the effect of chronic disease: cross-sectional questionnaire study. JMIR Aging.

[27] Wong CK, Yeung DY, Ho HC, Tse KP, Lam CY (2014). Chinese older adults' Internet use for health information. J Appl Gerontol.

[28] Ma Q, Chan AH, Teh PL (2021). Insights into older adults’ technology acceptance through meta-analysis. Int J Hum Comput Interact.

[29] Hedges LV, Olkin I (2014). Statistical Methods for Meta-Analysis.

[30] Borenstein M, Hedges LV, Higgins JP, Rothstein HR (2010). A basic introduction to fixed-effect and random-effects models for meta-analysis. Res Synth Methods.

[31] Balduzzi S, Rücker G, Schwarzer G (2019). How to perform a meta-analysis with R: a practical tutorial. Evid Based Ment Health.

[32] Schwarzer G, Carpenter JR, Rücker G (2015). Meta-Analysis with R.

[33] Quaosar GM, Hoque MR, Bao Y (2018). Investigating factors affecting elderly's intention to use m-Health services: an empirical study. Telemed J E Health.

[34] Pate L (2022). Factors affecting older adults’ adoption of wearable health-related internet of things applications [Dissertation]. Capella University.

[35] Techatraiphum V, Tharnuraikun A, Krathu W, Chutimaskul W (2016). Telemedicine acceptance framework for the elderly in Thailand. Proceedings of the 2016 International Conference on Information and Communication Technology Convergence.

[36] Boontarig W, Chutimaskul W, Chongsuphajaisiddhi V, Papasratorn B (2012). Factors influencing the Thai elderly intention to use smartphone for e-health services. Proceedings of the 2012 IEEE Symposium on Humanities, Science and Engineering Research.

[37] Cristescu I, Iordache DD, Tirlea C (2022). Behavioral intention to use smartwatches: a case study. Proceedings of the 14th International Conference on Electronics, Computers and Artificial Intelligence.

[38] Diño MJ, de Guzman AB (2014). Using partial least squares (PLS) in predicting behavioral intention for telehealth use among Filipino elderly. Educ Gerontol.

[39] Akhter H, Hossain A (2022). Investigating the determinants of mobile health apps adoption among elderly citizens in Bangladesh. Am Int J Bus Manag Stud.

[40] Alsswey A, Al-Samarraie H (2019). Elderly users’ acceptance of mHealth user interface (UI) design-based culture: the moderator role of age. J Multimodal User Interfaces.

[41] Cimperman M, Makovec Brenčič M, Trkman P (2016). Analyzing older users' home telehealth services acceptance behavior-applying an Extended UTAUT model. Int J Med Inform.

[42] Etemad-Sajadi R, Gomes Dos Santos G (2019). Senior citizens' acceptance of connected health technologies in their homes. Int J Health Care Qual Assur.

[43] Greer DB, Abel WM (2022). Exploring feasibility of mHealth to manage hypertension in rural black older adults: a convergent parallel mixed method study. Patient Prefer Adherence.

[44] Hoque R, Sorwar G (2017). Understanding factors influencing the adoption of mHealth by the elderly: an extension of the UTAUT model. Int J Med Inform.

[45] Hsiao CH, Tang KY (2015). Examining a model of mobile healthcare technology acceptance by the elderly in Taiwan. J Glob Inf Tech Manag.

[46] Jeng MY, Pai FY, Yeh TM (2022). Antecedents for older adults' intention to use smart health wearable devices-technology anxiety as a moderator. Behav Sci (Basel).

[47] Kim U, Chung T, Park E (2022). Quality characteristics and acceptance intention for healthcare kiosks: perception of elders from South Korea based on the extended technology acceptance model. Int J Environ Res Public Health.

[48] Li J, Ma Q, Chan AH, Man S (2019). Health monitoring through wearable technologies for older adults: smart wearables acceptance model. Appl Ergon.

[49] Li W, Shen S, Yang J, Tang Q (2021). Internet-based medical service use and eudaimonic well-being of urban older adults: a peer support and technology acceptance model. Int J Environ Res Public Health.

[50] Ma Y, Luo M (2022). Older people's intention to use medical apps during the COVID-19 pandemic in China: an application of the Unified Theory of Acceptance and Use of Technology (UTAUT) model and the Technology of Acceptance Model (TAM). Ageing Soc.

[51] Mascret N, Temprado JJ (2023). Acceptance of a mobile telepresence robot, before use, to remotely supervise older adults' adapted physical activity. Int J Environ Res Public Health.

[52] Mascret N, Delbes L, Voron A, Temprado J, Montagne G (2020). Acceptance of a virtual reality headset designed for fall prevention in older adults: questionnaire study. J Med Internet Res.

[53] Mukherjee S (2021). A quantitative study using the UTAUT2 model to evaluate the behavioral intention to use telemedicine via IoT-enabled devices by old [Dissertation]. University of the Cumberlands.

[54] Pal D, Funilkul S, Charoenkitkarn N, Kanthamanon P (2018). Internet-of-things and smart homes for elderly healthcare: an end user perspective. IEEE Access.

[55] Pywell JT (2021). Understanding the psychosocial drivers of adoption and use of mobile mental health interventions among older adults [Dissertation]. Northumbria University.

[56] Ren Z, Zhou G (2023). Analysis of driving factors in the intention to use the virtual nursing home for the elderly: a modified UTAUT model in the Chinese context. Healthcare (Basel).

[57] Rój J (2022). What determines the acceptance and use of eHealth by older adults in Poland?. Int J Environ Res Public Health.

[58] Talukder MS, Sorwar G, Bao Y, Ahmed JU, Palash MA (2020). Predicting antecedents of wearable healthcare technology acceptance by elderly: a combined SEM-Neural Network approach. Technol Forecast Soc Change.

[59] Tsai TH, Lin WY, Chang YS, Chang PC, Lee MY (2020). Technology anxiety and resistance to change behavioral study of a wearable cardiac warming system using an extended TAM for older adults. PLoS One.

[60] Tu CK, Liu H (2021). The moderating effects of subjective well-being on the elderly's acceptance and use of gerontechnology: an extended UTAUT model. Proceedings of the 5th International Conference on Management Engineering, Software Engineering and Service Sciences.

[61] Wang X, Lee CF, Jiang J, Zhu X (2023). Factors influencing the aged in the use of mobile healthcare applications: an empirical study in China. Healthcare (Basel).

[62] Xu W, Liang HN, Yu K, Wen S, Baghaei N, Tu H (2022). Acceptance of virtual reality exergames among Chinese older adults. Int J Hum Comput Interact.

[63] Zhang X (2023). Motivational factors influencing intention to use mobile health in older adults: an integrated model of the technology acceptance model and uses and gratifications theory [Dissertation]. University of Nottingham Ningbo.

[64] Zin KS, Kim S, Kim H, Feyissa IF (2023). A study on technology acceptance of digital healthcare among older Korean adults using extended tam (extended technology acceptance model). Adm Sci.

[65] Chen K, Chan AH (2014). Gerontechnology acceptance by elderly Hong Kong Chinese: a senior technology acceptance model (STAM). Ergonomics.

[66] Hedman A, Lindqvist E, Nygård L (2016). How older adults with mild cognitive impairment relate to technology as part of present and future everyday life: a qualitative study. BMC Geriatr.

[67] Levine DM, Lipsitz SR, Linder JA (2018). Changes in everyday and digital health technology use among seniors in declining health. J Gerontol A Biol Sci Med Sci.

[68] Lee C, Coughlin JF (2014). PERSPECTIVE: older adults' adoption of technology: an integrated approach to identifying determinants and barriers. J Prod Innov Manage.

[69] Hauk N, Hüffmeier J, Krumm S (2018). Ready to be a silver surfer? A meta-analysis on the relationship between chronological age and technology acceptance. Comput Human Behav.

[70] Goswami A, Dutta S (2016). Gender differences in technology usage—a literature review. Open J Bus Manag.

[71] McCoy S, Galletta DF, King WR (2017). Applying TAM across cultures: the need for caution. Eur J Inf Syst.

[72] Demiris G, Rantz MJ, Aud MA, Marek KD, Tyrer HW, Skubic M, Hussam AA (2004). Older adults' attitudes towards and perceptions of "smart home" technologies: a pilot study. Med Inform Internet Med.

[73] Pang NQ, Lau J, Fong SY, Wong CY, Tan KK (2022). Telemedicine acceptance among older adult patients with cancer: scoping review. J Med Internet Res.

[74] Ahmad A, Mozelius P (2022). Human-computer interaction for older adults - a literature review on technology acceptance of eHealth systems. J Eng Res Sci.

[75] Yu-Huei C, Ja-Shen C, Ming-Chao W (2019). Why do older adults use wearable devices: a case study adopting the senior technology acceptance model (stam). Proceedings of the 2019 Portland International Conference on Management of Engineering and Technology.

[76] Hirunyawipada T, Paswan AK (2006). Consumer innovativeness and perceived risk: implications for high technology product adoption. J Consum Mark.

[77] Classen S, Mason J, Wersal J, Sisiopiku V, Rogers J (2020). Older drivers' experience with automated vehicle technology: interim analysis of a demonstration study. Front Sustain Cities.

[78] Yang HJ Meta-analysis data Final(JMIR). figshare.

