# Supplementary material for: Factors Influencing Health Care Technology Acceptance in Older Adults Based on the Technology Acceptance Model and the Unified Theory of Acceptance and Use of Technology: Meta-Analysis
Source: J Med Internet Res. 2025 Mar 28;27:e65269. doi: 10.2196/65269 (PMC11992498; doi:10.2196/65269)

**Supplementary File**

**(Search term)**

(("older adults" OR "elderly" OR "ageing" OR "aging")

AND

("unified theory of acceptance and use of technology" OR "UTAUT" OR "technology acceptance model" OR "TAM" OR "acceptance" OR "adoption" OR "intention")

AND

("health" OR "healthcare" OR "well-being" OR "gerontechnology"))

**(Additional Figures)**

Forest plot of PU-BI


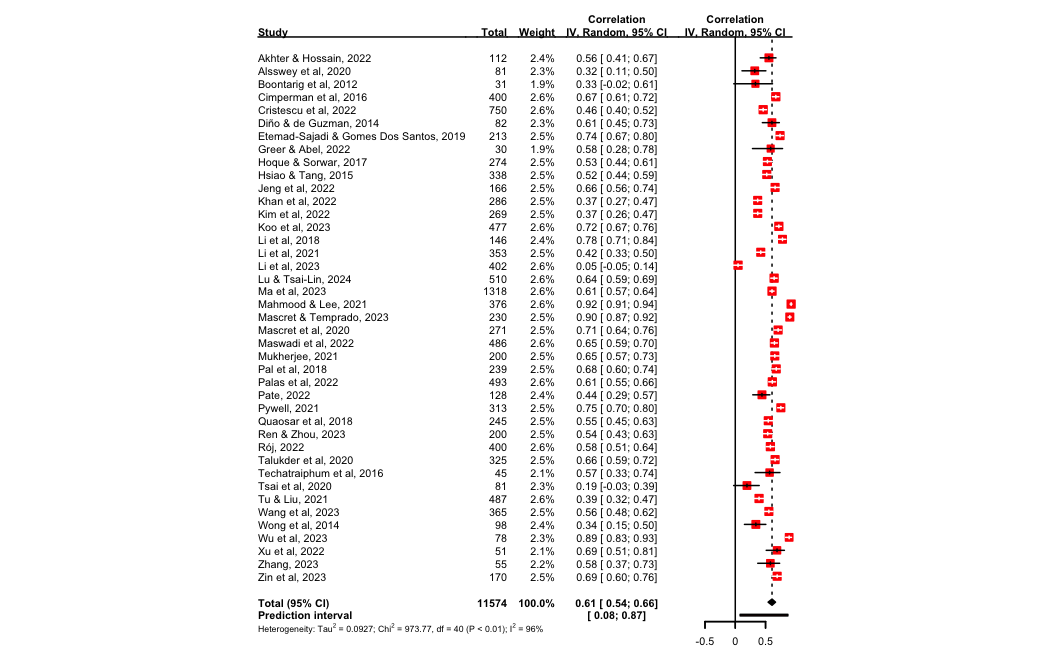


Forest plot of PEOU-BI


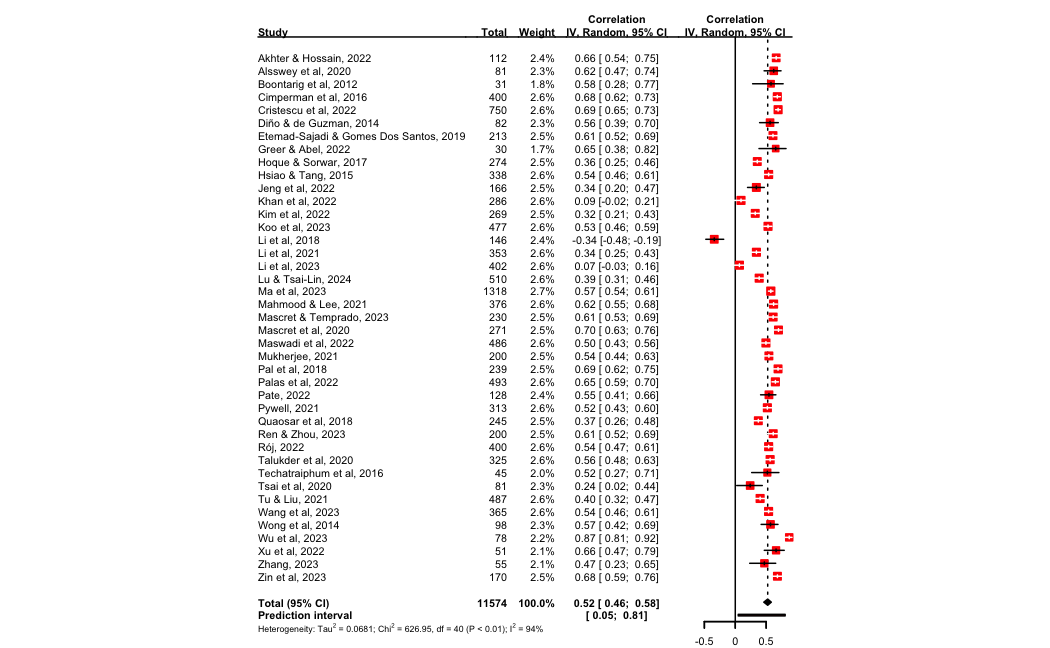


Forest plot of SI-BI


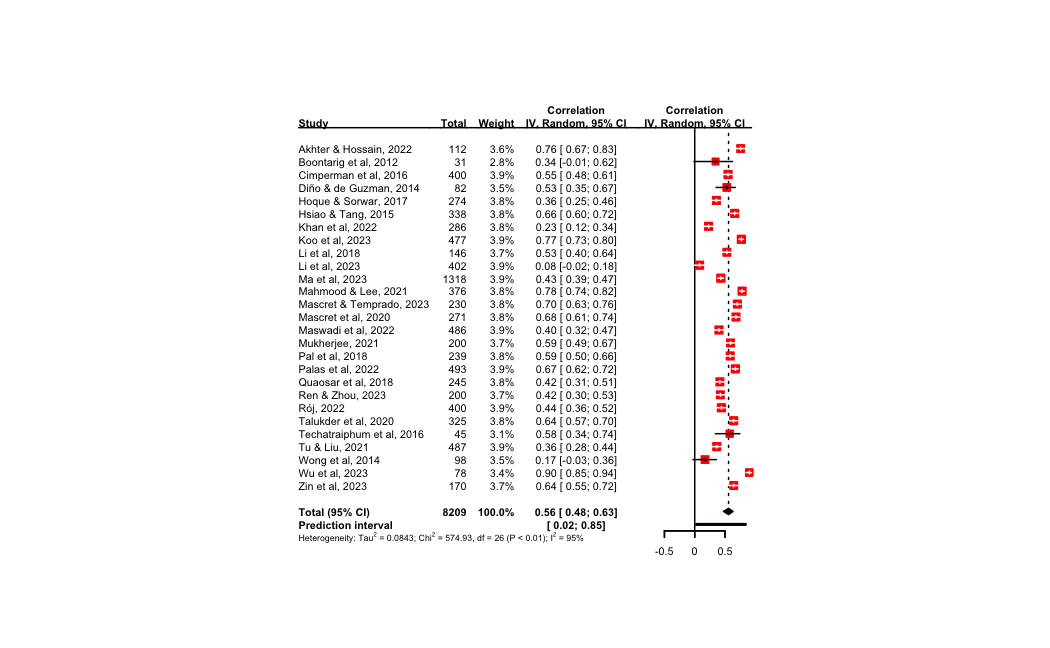


Bubble plot of meta regression of gender ratio for PU-BI


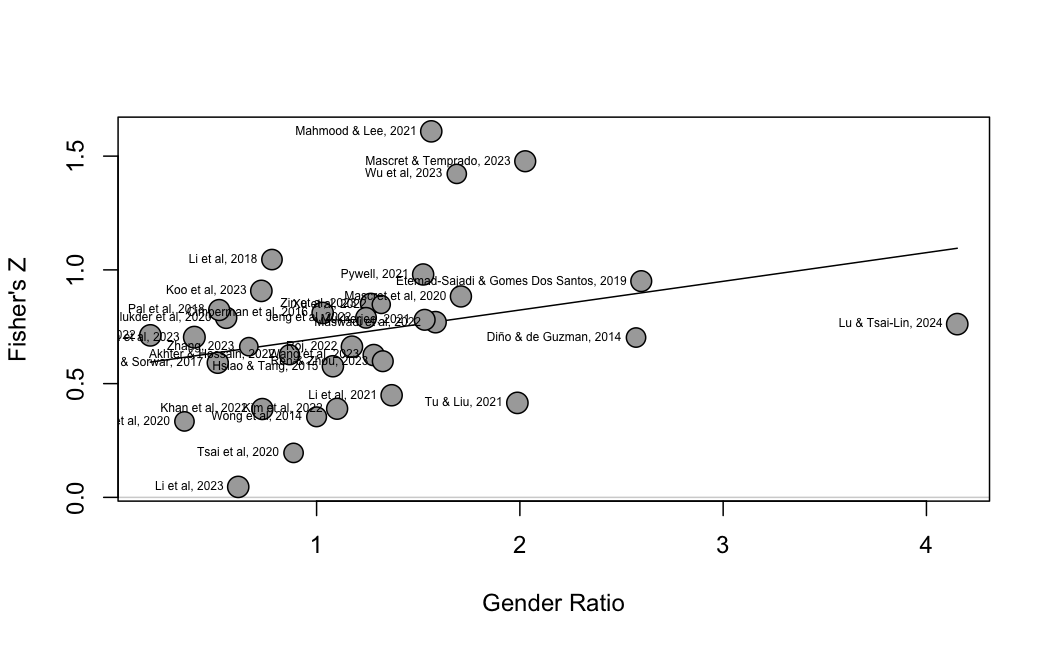


Bubble plot of meta regression of gender ratio for PEOU-BI


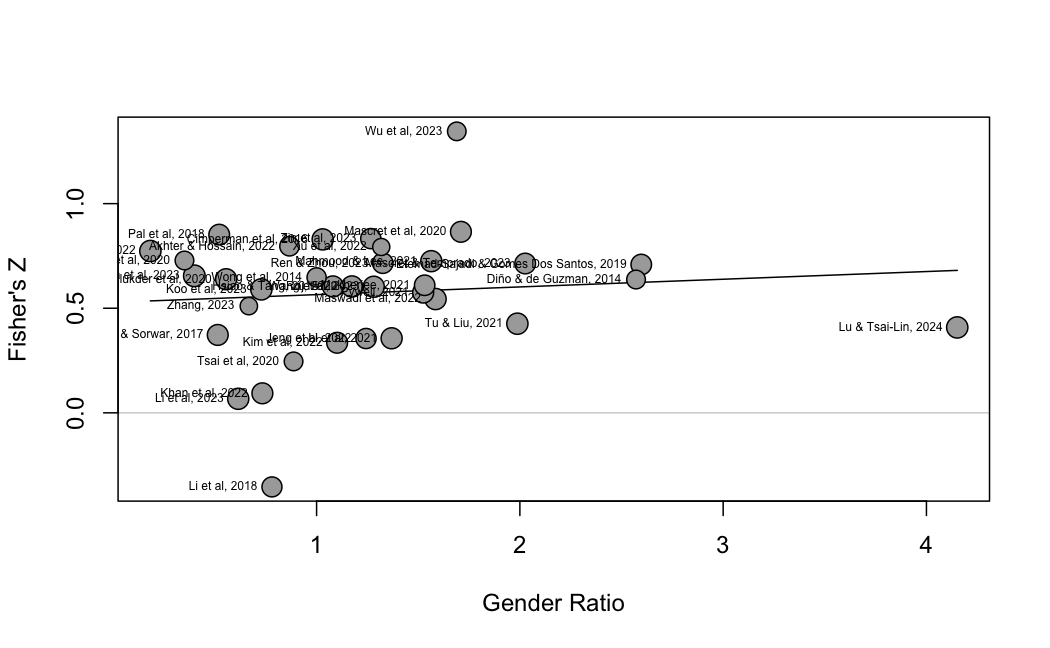


Bubble plot of meta regression of gender ratio for SI-BI


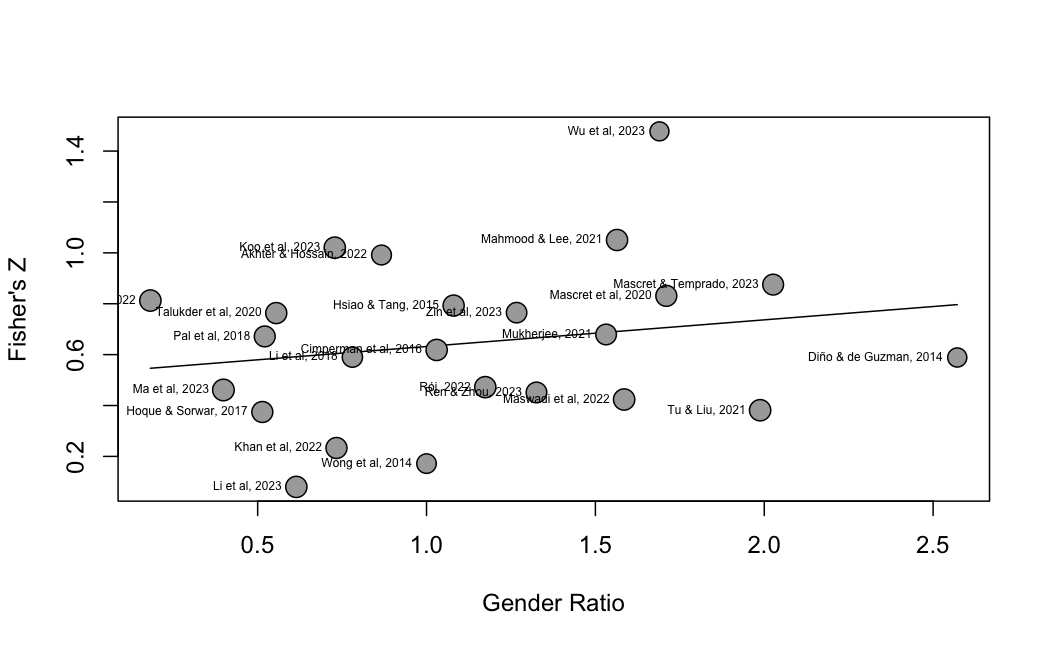


Bubble plot of meta regression of mean age of sample for PU-BI


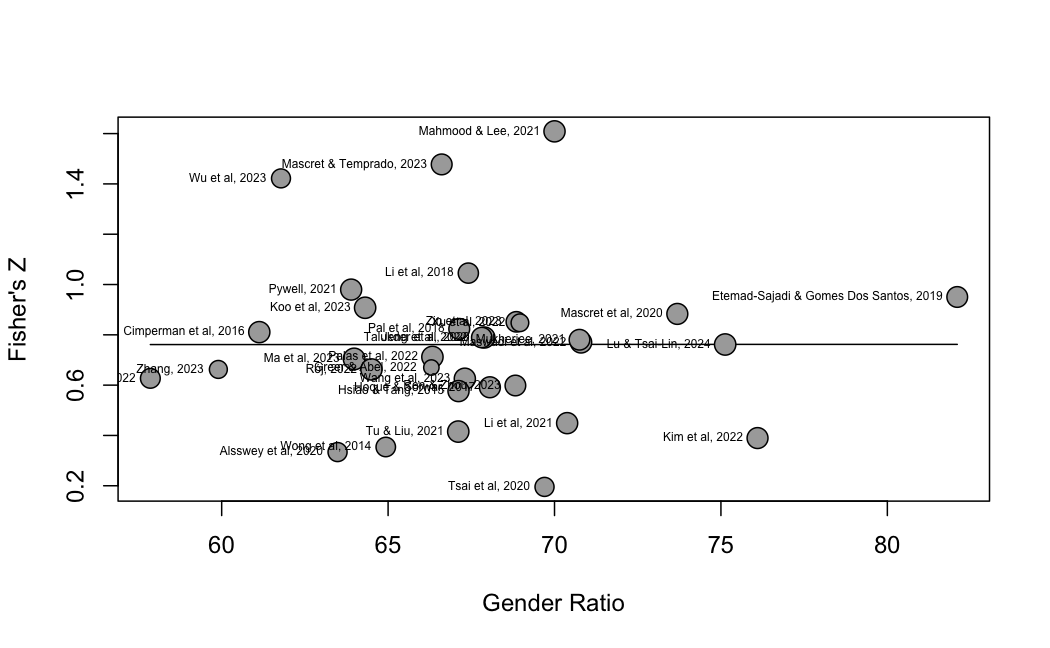


Bubble plot of meta regression of mean age of sample for PEOU-BI


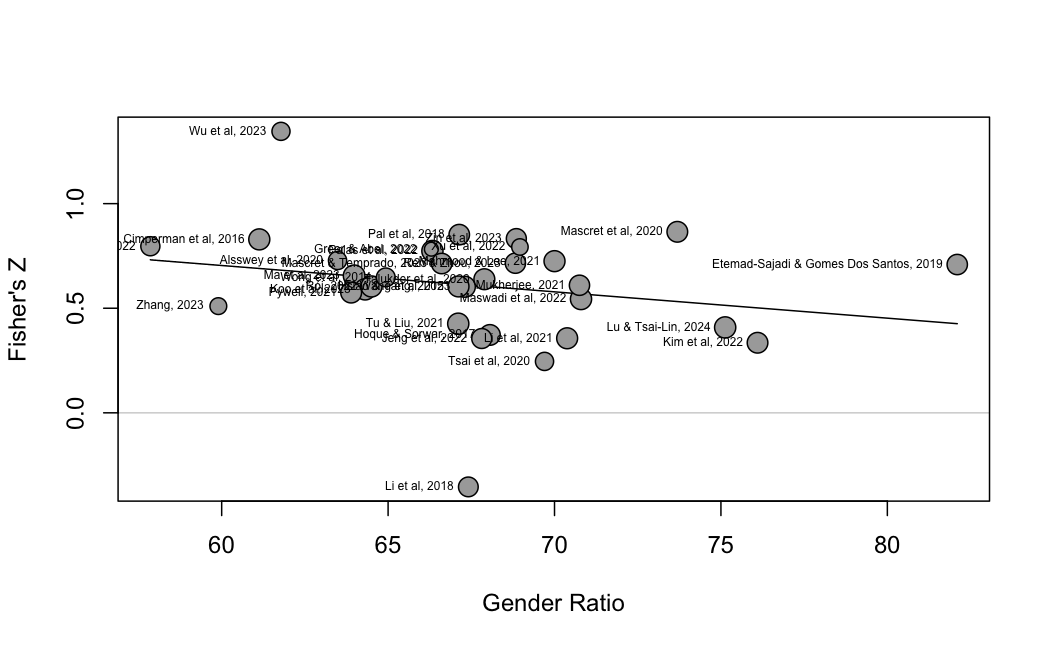


Bubble plot of meta regression of mean age of sample for SI-BI


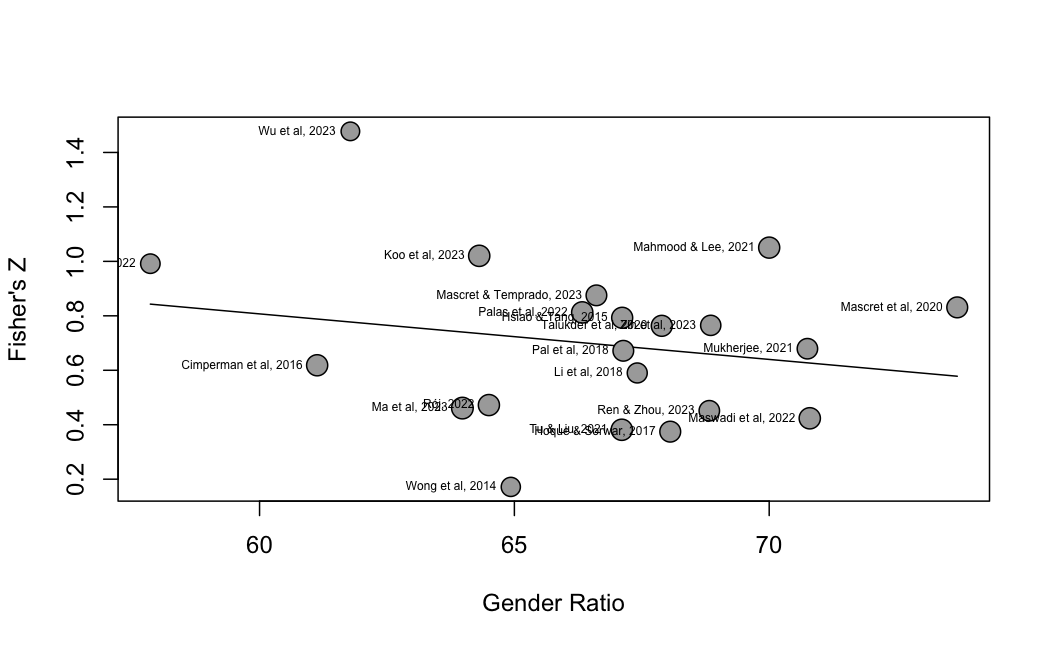


Forest plot of subgroup analysis of region for PU-BI


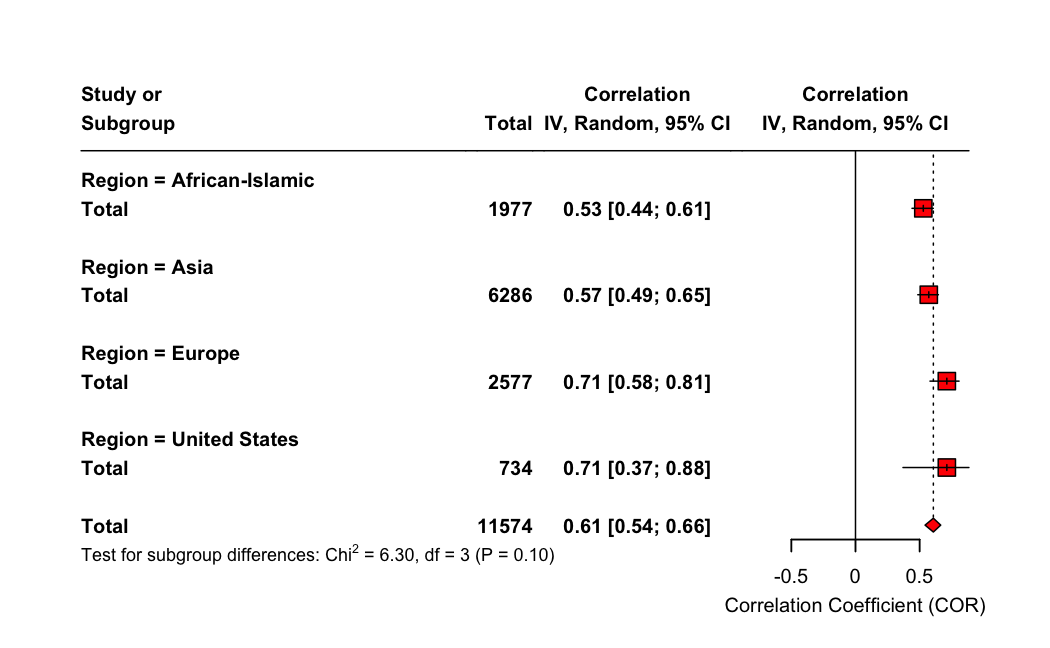


Forest plot of subgroup analysis of region for SI-BI


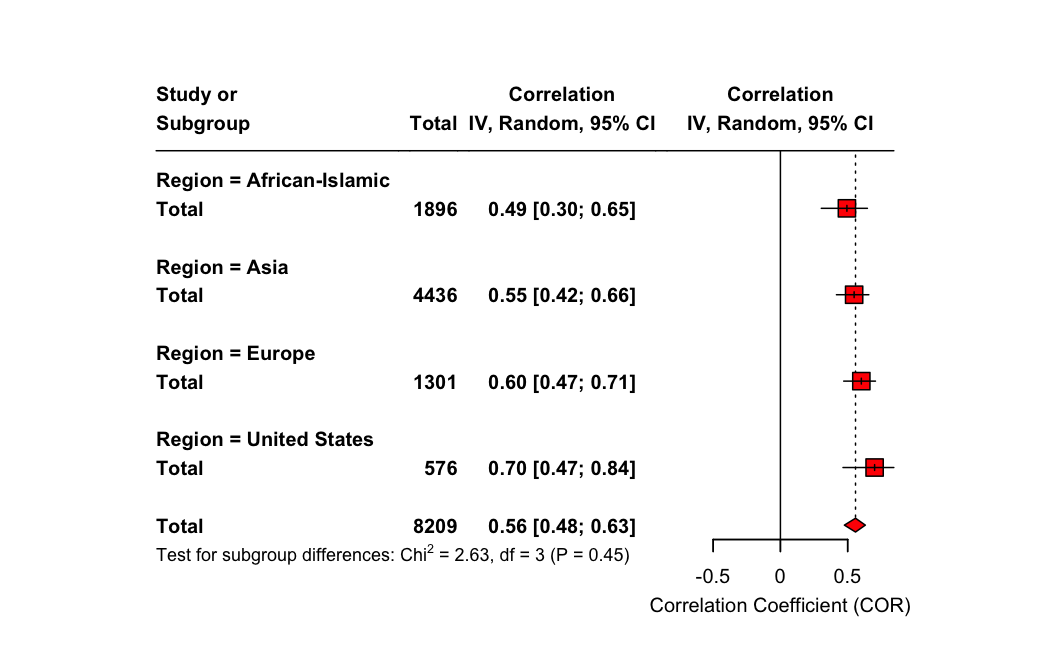


Forest plot of subgroup analysis of type of healthcare technology for SI-BI


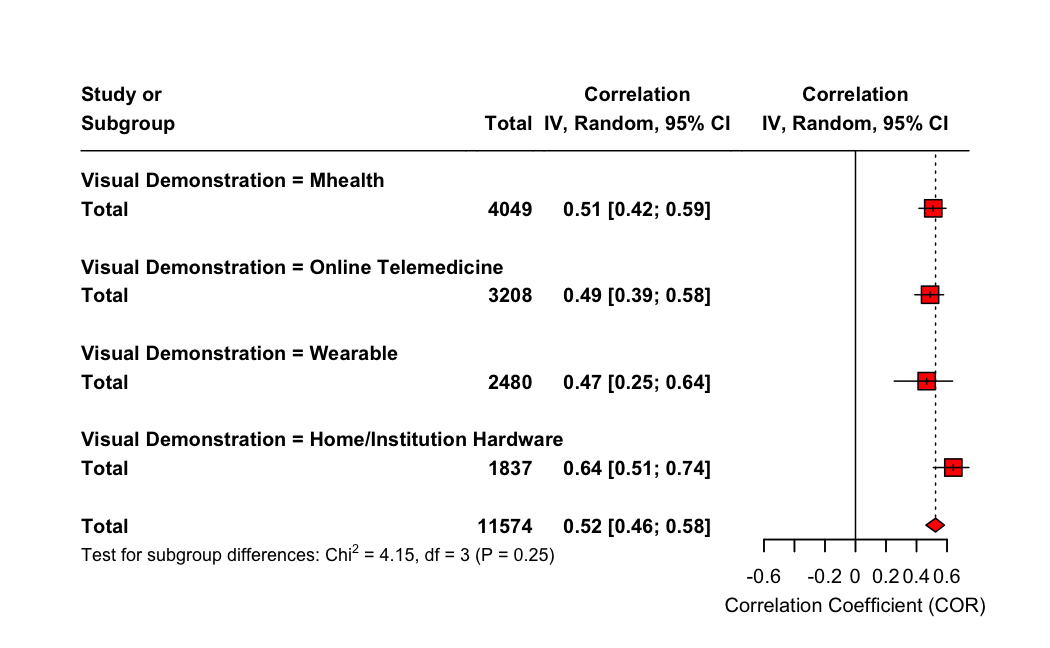


Forest plot of subgroup analysis of presence of visual demonstration for SI-BI


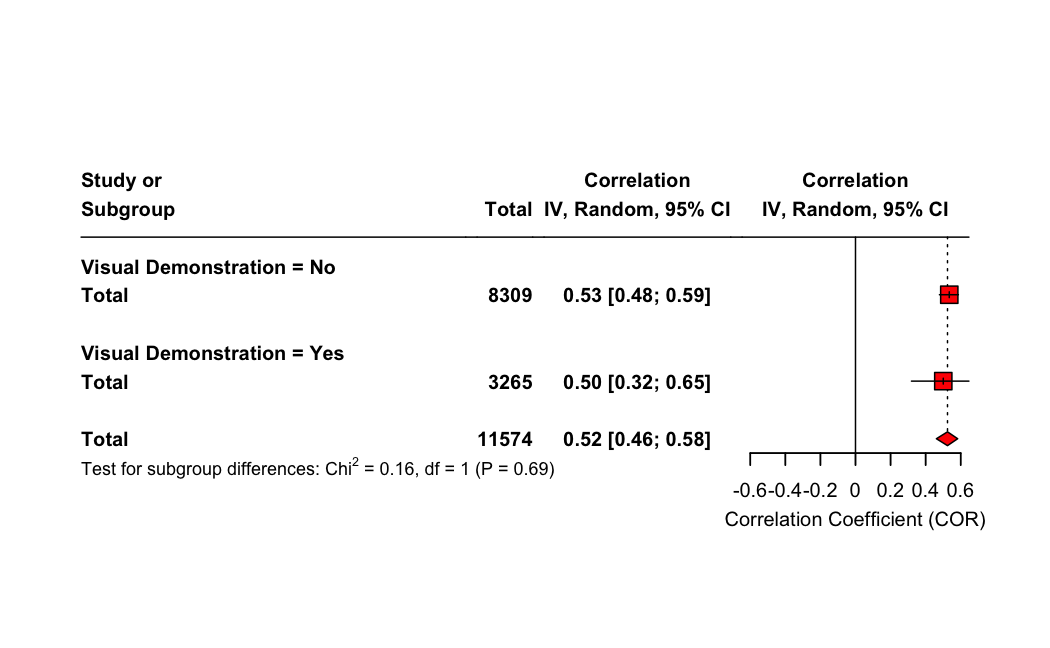

Supplement: Multimedia Appendix 1 [file jmir_v27i1e65269_app1.docx]
